# 5D proteomic approach for the biomarker search in plasma: Acute myeloid leukaemia as a case study

**DOI:** 10.1038/s41598-017-16699-2

**Published:** 2017-11-27

**Authors:** Syed Kashif Raza, Mahwish Saleem, Tahir Shamsi, M. Iqbal Choudhary, Atta-ur-Rahman, Syed Ghulam Musharraf

**Affiliations:** 1 Dr. Panjwani Center for Molecular Medicine and Drug Research, International Center for Chemical and Biological Science, University of Karachi, Karachi, 75270 Pakistan; 2 H.E.J. Research Institute of Chemistry, International Center for Chemical and Biological Science, University of Karachi, Karachi, 75270 Pakistan; 3 Department of Biochemistry, Faculty of Sciences, King Abdulaziz University, Jeddah, 21412 Saudi Arabia; 4 National institute of Blood Diseases, Karachi, Pakistan

## Abstract

Acute myeloid leukaemia (AML) is a type of cancer affecting all ages but it is more common in adults, as compared to children. Recent advancements in proteomics and mass spectrometry tools, offer a comprehensive solution to study the molecular complexity of diseases, such as cancers. This study is focused on the proteomic profiling of AML in comparison to healthy control for which, a systematic 5D proteomic approach for the fractionation of pooled plasma samples was used. Methodology includes depletion of Top-7 abundant proteins, ZOOM-isoelectric focusing (ZOOM-IEF), two-dimensional gel electrophoresis (2-DGE), and matrix-assisted laser desorption/ionization mass spectrometry (MALDI-MS) analysis followed by the validation of identified biomarker proteins using enzyme linked immunosorbent assay (ELISA). Up-/down-fold changes in concentration of proteins were observed in 2-DGE of AML in comparison with the healthy control and a total of 34 proteins were identified in fractioned plasma. Among them, fifteen proteins were significantly differentiated and five proteins; SAA1, complement factor C7, ApoE, plasminogen, and ApoA1 were later verified by ELISA in individual samples, which showed that SAA1 and plasminogen could be used as potential biomarker for AML.

## Introduction

Acute myeloid leukaemia (AML) is an aggressive blood disease comprising of many subtypes with the dominant features of infiltration and accumulation of myeloid lineage hematopoietic blast cells in bone marrow and peripheral blood^1,2^. It is common in adults, and its incidence rate increases with increase in age however, also affects small percentage of children (10–15%)^3^. Though 40–50% of the older patients with AML achieve complete remission, but 5-years survival rate in patients above 65 years is 5% due to increased rate of relapse^1^. Sub-classification is based on cellular morphology, hematopoietic lineage as well as common translocations and mutations^4^. Among cytogenetic abnormalities, the most common abnormality is internal tandem repeats of the FLT-3 gene. Some cases show chromosomal translocation inv(16) and t(8; 21) generating fusion protein which involve the core binding factor (CBF) genes. These, together with t(15; 17) variant show good prognosis. Many cases of AML also carry mutations in the nucleoplasmin (NPM) gene. Though different AML subtypes are considered as separate genetic diseases, but they can be grouped together because usually their treatment and prognosis are similar. Clinical features include low platelets count and increased tendency to bleed along with disseminated intravascular coagulation. In advanced cases, metastasis to tissues, such as skin, gums, and central nervous system tissues also occurs. Prognosis of patients and planning of treatment largely depends on cytogenetic and molecular analysis. Treatment strategy for AML is both supportive and specific involving the use of intensive and selective chemotherapy. Another treatment option is stem cell transplant which minimizes the rate of relapse but adds further toxicity, because of the treatment regime^5^.

Proteomic profiling using various approaches including mass spectrometry are being effectively used as a powerful tool for the understanding of diseases and identification of biomarkers particularly in cancers^6^. However, identification of reliable and sensitive protein biomarker depends on many factors specially the proteomic fractionation strategy being applied. A number of studies based on mass spectrometry, combining proteomic technologies such as two-dimensional gel electrophoresis (2-DGE), difference gel electrophoresis (DIGE), immunoprecipitation, and affinity chromatography in combination with matrix assisted laser desorption ionization-time of flight mass spectrometry (MALDI-TOF MS), surface-enhanced laser desorption/ionization time-of-flight (SELDI-TOF) mass spectrometry, liquid chromatography–mass spectrometry (LC-MS) and shotgun proteomics have been investigated for the proteomic profiling of AML to reveal new insights and diagnostic biomarkers^7–12^.

Our key objective in this study is to identify the proteomics based biomarkers for AML in comparison to healthy control using 5D protein strategy *i.e*. depletion of most abundant proteins using fast protein liquid chromatography (FPLC), ZOOM-IEF, 2-DGE, and MALDI-MS analysis followed by the validation of identified biomarker proteins through ELISA. In-solution isoelectric focusing (IEF) helps in the detection of low abundant proteins and improves the detection range because large amounts of proteins of specific pH can be applied to narrow pH range gel^13^. An early study has evaluated the technical use of microscale solution-phase IEF after immunodepletion of human proteome organization (HUPO) plasma, combined with narrow range 2-DGE, and stated to be effective and reproducible^14^. Therefore, it is expected that this developed strategy will be useful for the quest of proteomic biomarkers in other diseases as well. Moreover, similar fractionation strategy combining with MALDI-MS has already been used for the biomarker profiling of renal cancer patients^15^.

## Experimental

### Auxological data of subjects

A total of fasting 50 plasma samples of AML were included in this study. The subtypes of AML which were included in this study are listed in Table S1. A total of 50 healthy male and 50 female healthy volunteers were also selected for the study (Table S2). All healthy individuals were free of AML, and other haematological diseases, and no previous familial history of AML. At the time of sampling, the physical condition was good, with normal vital signs and no history of any other disease.

### Sample collection and processing

The study was approved by the Independent Ethics Committees, National Institute of Blood Diseases & Bone Marrow Transplantation Hospital, Karachi, and the International Centre for Chemical and Biological Sciences (ICCBS), University of Karachi, Pakistan. All samples were collected according to approved protocol; a written informed consent and a thorough questionnaire was filled by every patient and healthy volunteer. Sample collection was carried out in accordance with relevant guidelines and regulations. Individual blood samples were collected, processed, and stored according to the human proteome organization (HUPO) standard protocol^16^. Human blood (5 mL) was transferred by venepuncture into evacuated blood collection tubes, containing K_2_-ethylenediaminetetra acetic acid (K_2_-EDTA). The plasma was separated by centrifugation at 2,200 × g for 10 min at 4 °C. Pool of individual samples were used in this study, because pooling is cost effective, include greater statistical power, improved ability to compare results and validation of models. Pooled plasma has already been applied in many studies^17–20^. To make healthy pool, equal volumes of each individual healthy plasma sample ware mixed to obtain the healthy Pakistani pooled plasma. Similarly, AML samples were also pooled by combining equal amounts of every AML specimen. Pooled plasma samples were then subjected to aliquoting, and stored at −80 °C until further processing.

### 1D SDS-PAGE analysis of samples

One-dimensional sodium dodecyl sulphate polyacrylamide gel electrophoresis (1D SDS-PAGE) was performed for comparative analysis of healthy and diseased samples on X Cell SureLock system (Invitrogen). Chemicals and reagents for 1D SDS-PAGE were purchased from Invitrogen (USA). β-mercaptoethanol, sucrose, and Tris HCL were purchased from Sigma Aldrich (USA).

### Depletion of abundant proteins through MARS column

Depletion of top seven most abundant proteins was carried out using Multiple Affinity Removal Column (MARS) Hu-7 (4.6 × 50 mm), purchased from Agilent (USA) on ÄKTA™ FPLC system (GE Healthcare, Sweden). The column has an affinity for the albumin, IgG, IgA, transferrin, antitrypsin, haptoglobin (HPT), and fibrinogen in plasma. Protease inhibitors; ethylene diamine tetra-acetic acid (EDTA), leupeptin, pepstatin-A, and phenyl methanesulphonyl flouride (PMSF) were purchased from Sigma Chemicals (USA). 500 mL Vacuum Filter/Storage Bottle System, 0.22 µm was purchased from Corning (USA). The plasma sample (300 µL) was depleted according to the kit protocol. The plasma amount used was according to the column capacity, and required sample size for multiple replicate depletions. 1D SDS-PAGE analysis of all fractions, including bound and unbound fractions was performed to check the sample recovery after depletion. Unbound fractions were pooled and concentrated using 5 kDa molecular weight cut off (MWCO) tubes for both pools. For concentrating the pooled bound fractions, the sample was centrifuged several times at 3,500 rpm for 15 min at 4 °C to obtain an amount of 200 µL. Enrichment efficiency was checked by loading the equivalent to 0.1 µL plasma from the fractions before concentrating and after concentrating.

### Reduction and alkylation

Urea was purchased from Invitrogen (USA), tris(hydroxymethyl)aminomethane (Tris) from Boehringer Mannheim (Germany), dichlorodiphenyltrichloroethane (DTT) and iodoacetamide (IAM) from SERVA (Germany). Acetone, and trichloroacetic acid (TCA) were purchased from Fisher Scientific (UK) and Scharlau (Spain), respectively. For denaturation and pH adjustment, concentrated depleted sample was adjusted to 8 M urea and 20 mM Tris. To reduce the proteins, the sample was adjusted to 20 mM DTT, and subsequently for alkylation, to 50 mM IAM. To quench the alkylation, the sample was adjusted to 1% DTT. Protein precipitation was carried out using TCA/acetone protocol with some modifications^21^. Sample, ice-cold acetone and 100% TCA solution, were mixed and placed at −20 °C for 1 hr. to precipitate the proteins. The tube was then centrifuged at 5,000 rpm for 30 min to have the proteins pelleted down. The pellet was washed two to three times with ice-cold acetone.

### ZOOM-IEF

The ZOOM-IEF® Fractionator Combo Kit including ZOOM-IEF fractionator was purchased from Invitrogen (USA). The sample pellet was dissolved in ZOOM buffer by vortexing for 5 min, followed by the addition of 26 µL of ZOOM carrier ampholytes (pH 3–10) along with small amount of bromophenol blue. To remove the fine particles, the sample was centrifuged at 12,000 rpm for 1 min through 0.22 µm spin filter. Fractionation was performed according to established protocol^22^. 1D SDS-PAGE analysis of all five ZOOM fractions was performed to check the efficiency of IEF, and to compare the samples from healthy persons with AML patients.

### 2-D gel electrophoresis

2-DGE was performed on Bio-Rad PROTEAN IEF cell. The ReadyPrep 2-D Starter Kit, ReadyStrip IPG Strips, Ready Gel precast gel, mineral oil, 10X Tris/glycine/SDS Buffer, and paper wicks were purchased from Bio-Rad (USA). Buffers were prepared according to the kit protocol. 125 µL of freshly prepared rehydration/sample buffer for 7 cm IPG strip was added in a conical centrifuge tube containing the sample pellet, and vortexed to dissolve the pellet. The sample was centrifuged to settle down the fine particles. The whole procedure was performed according to the kit protocol. Gel images were taken through Gel DOC 800 system (Bio-Rad, USA).

### Mass spectrometric analysis

Analysis was performed using MALDI-TOF-TOF MS (Ultraflex III, Broker Daltonics Germany). Mass spectrometric profile was obtained by flexAnalysis version 3.0. (Bruker Daltonics). The protein spots of interest were extracted from the stained gels using the manual cutting procedure and digested according to formerly mentioned protocol^23,24^.

The digested peptides were analysed using standard protocol^25^. Briefly, the samples were mixed with equal amounts of freshly prepared α-cyano-4-hydroxycinnamic acid in acetonitrile (ACN) in 1:1 ratio. Calibration of MALDI-TOF was carried out in the reflector positive mode using peptide calibrant standard I (Bruker Daltonics). A 337-nm nitrogen laser and a 2 GHz digitilizer were used. Mass spectra were obtained with 25 KV of ion acceleration, 6 KV lens potential, and high gating strength to deflect ions with a mass below 500 *m/z* values. Spectra were obtained in the mass range of 500–3000. Every spectrum was the sum of 2000 laser shots within the same spot (200 shots/position) and intensity of 20–40%.

### ELISA analysis

ELISA was performed for five proteins, serum amyloid A (SAA1), plasminogen, apolipoprotein E (ApoE), complement factor C7 and apolipoprotein A1 (ApoA1) on Thermo Fischer Scientific™ Multiskan™ FC Microplate Photometer (USA). ELISA kits were purchased from Crystalchem (USA) and Assaypro (USA). Plasma samples of 15 healthy individuals and 18 AML patients were diluted according to the kit protocol, using 1X diluent. Standard and diluted samples were dispensed into wells. After one-hour incubation, the samples were aspirated and the wells were washed 4 times using 1X wash buffer. The antibody-HRP conjugate was then applied for 20 min. On completion of this step, 4 times washing was carried out again using 1X wash buffer. The substrate solution was then added and left for 10 min for reaction completion. Stop solution was added and readings were taken at 450 and 630 nm. This procedure with some modifications according to the manufacturer’s protocol was applied to validate our results for selected proteins; complement factor C7, plasminogen, and ApoE, for which ELISA kits were purchased from Assaypro (USA).

### Statistics and data analysis

The analysis of 2-DGE images, detection of spots, spot matching, and semi-quantitative statistical analysis were performed using the Bio-Rad PDQuest version 8.0.1. Bio-Rad (USA). Master gel was used to compare the gels of healthy and AML pool. The analysis involved matching the gels, differences and similarities in spots pattern, background subtraction, and removal of artefacts (horizontal and vertical streaks). *T*-test was used to study the differential protein expression among protein spots from healthy and AML pool gels, with *p* < 0.05 and four-fold change in spot intensity was selected as threshold. After automated matching, the detected spots were manually edited for greater accuracy.

After MALDI-MS the protein identification was carried out through MASCOT database search – Matrix Science, based on mass fingerprinting (PMF) using Swiss-Prot and NCBInr databases. Peptide modification which we have chosen as a fixed modification during the search, was carbamidomethylation of cystein. The oxidation of methionine was used as variable modification. The maximum number of missed cleavages was set to 1, peptide tolerance 100 ppm/1 Da, and p < 0.05 were used to identify proteins.

Identified proteins were further subjected to Gene Ontology (GO) based analysis to know the function. The connections between differently expressed proteins with each other and their connections with the other proteins were assessed by the STRING: EMBL (European Molecular Biology Laboratory) software^26^. Minimum required interaction score of medium confidence 0.400 was used.

## Results

### Fractionation of plasma samples

**Figure 1:**
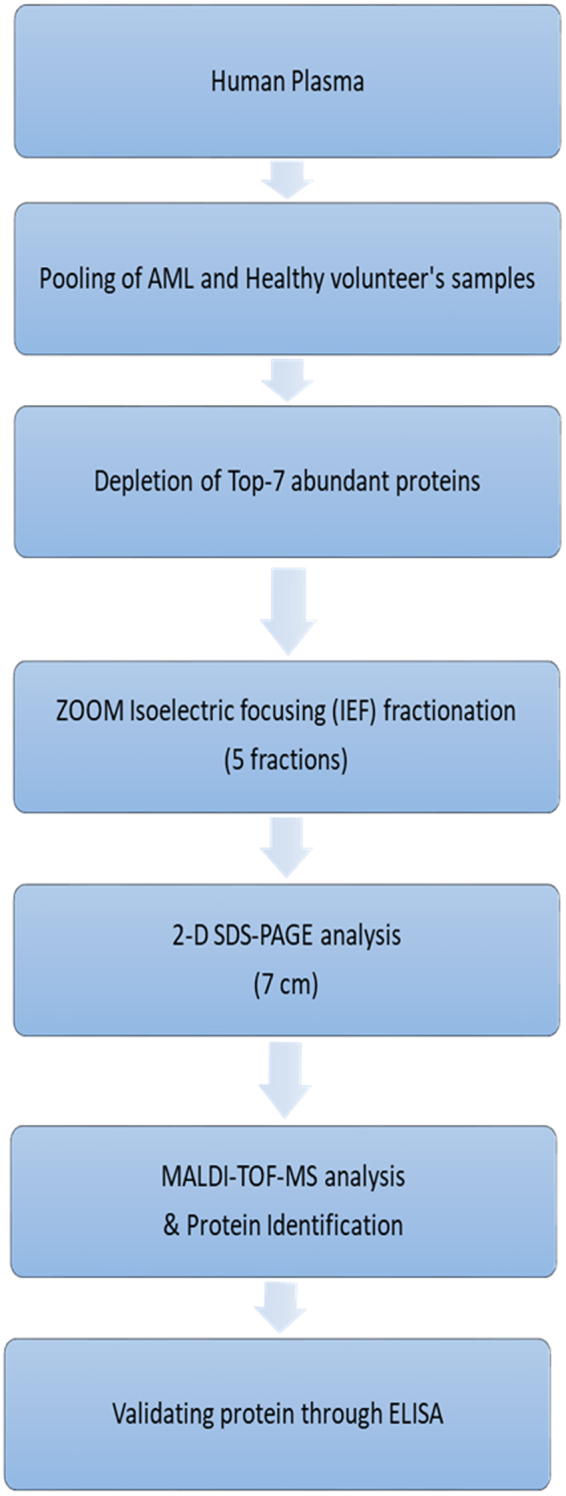
Scheme used for analysis of the human plasma sample.

Using a systematic strategy (as shown in Fig. 1) pool plasma samples of AML and healthy subjects were first depleted for the top-7 abundant proteins through a MARS column. The resultant FPLC spectrum showed a clear separation of unbound and bound fractions, and was in the agreement with the figure provided by the manufacturer (Fig. S1). The resultant low-abundant proteins in flow through were then analysed by 1D SDS-PAGE for depletion efficiency which showed an effective protein depletion (Fig. S2). After depletion, the unbound portion was concentrated using 5 kDa MWCO tubes, followed by enrichment efficiency checking by 1D SDS-PAGE and found to be acceptable (Fig. S3).

**Figure 2:**
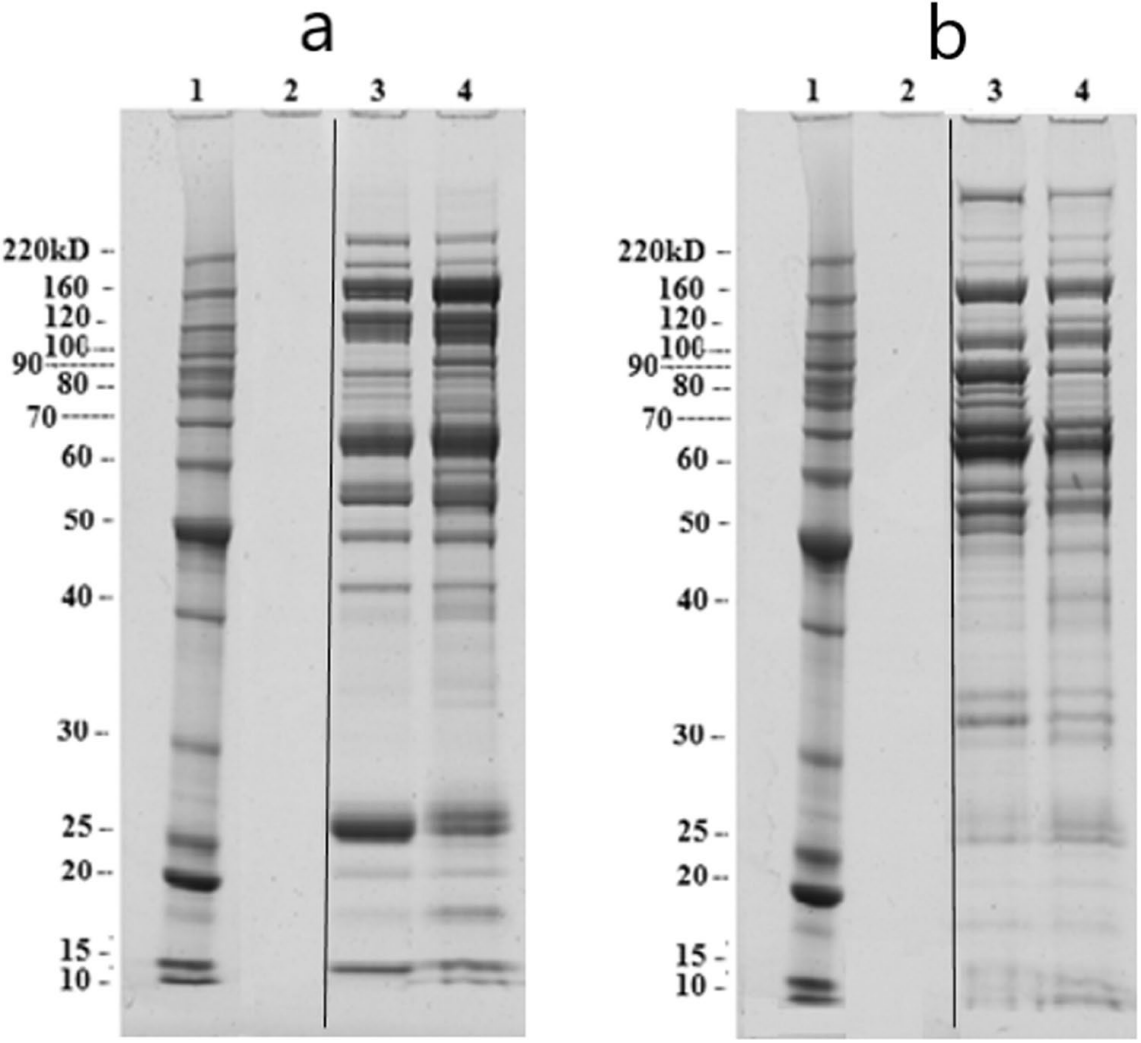
(**a**) Comparison of fraction-3 (pH: 5.4–6.2) after ZOOM_IEF. 1: Protein ladder, 2: Blank, 3: Healthy pool, 4: AML pool, (**b**) Comparison of fraction-4 (pH: 6.2–7.0) after ZOOM_IEF. 1: Protein ladder, 2: Blank, 3: Healthy pool, 4: AML pool. The amount of fractions loaded into gel was equivalent to 3 uL of original plasma.

The unbound portion, after enrichment by 5 kDa MWCO and protein precipitation, was further resolved by ZOOM-IEF over a pH range of 3.0 to 10 into five fractions of different pH ranges; pH: 3.0–4.6, pH: 4.6–5.4, pH: 5.4–6.2, pH: 6.2–7.0, and pH: 7.0–10.0 (Fig. S4). 1D SDS-PAGE analysis of all fractions showed that among all fractions two of pH: 5.4–6.2 and pH: 6.2–7.0 from AML and healthy samples pool had many protein bands with some differential pattern in the molecular weight range of 10–266 kDa. Therefore, these two fractions were subjected to further analysis. A comparative 1D SDS-PAGE picture of these two fractions from AML and healthy samples pool is shown in Fig. 2.

**Figure 3:**
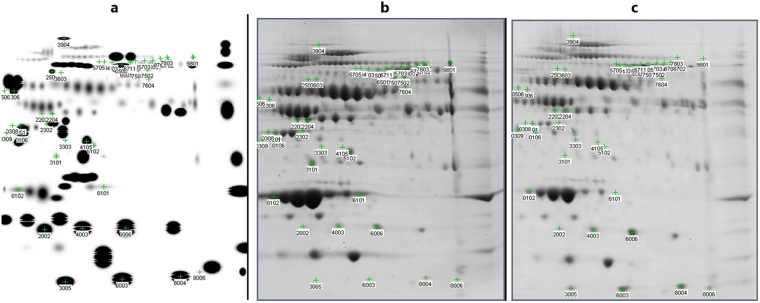
Comparison of AML pool and healthy pool 2-DGE image. Highlighted spot numbers are those who are more than 95% significant and quantity changes more than 4-fold, which were further analysed. (**a**) Master gel, created my adding spots from AML pool and healthy pool gel images using PDQuest software. (**b**) 2-DGE map of healthy pool in the range of pH 5.4–7.0. (**c**) 2-DGE map of AML pool in the range of pH 5.4–7.0.

**Figure 4:**
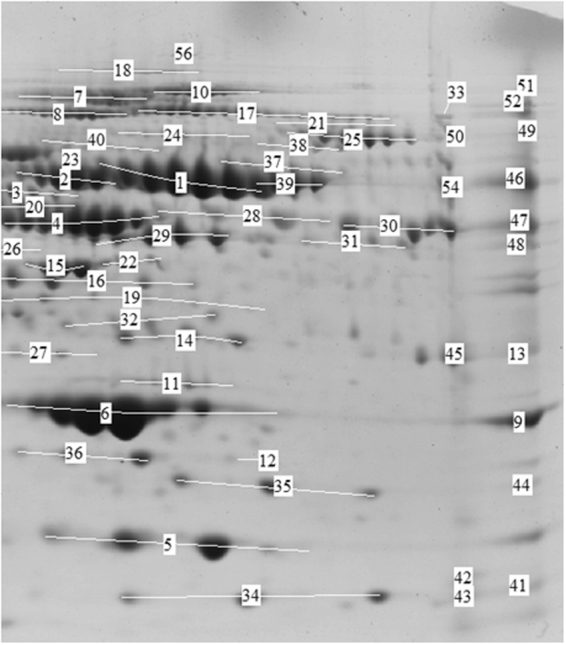
Identified variants and subunits of proteins in 2-DGE image through MALDI-MS and MASCOT database searching. [1, 39, 46: hemopexin, 2: alpha-1β-glycoprotein, 3, 20: kininogen-1, 4: Vit-D-binding protein, 5: transthyretin, 6, 9: apolipoprotein A-I, 7, 8, 52: ceruloplasmin, 10, 51, 55: complement factor H, 11: human serum amyloid-P component, 12, 13, 14, 32: apolipoprotein E, 15: apolipoprotein A-IV, 16, 35, 44: haptoglobin, 17, 53: alpha 2-macroglobulin, 18: fibronectin, 21: complement factor 7, 22: CD5 antigen-like, 23, 40: prothrombin, 24: complement C1r-subcomponent, 25: complement factor B, 26: complement C4-A, 27: alpha-1 microglobulin, 28, 29, 48: fibrinogen gamma chain, 30, 31, 47: fibrinogen beta chain, 33, 49, 50: plasminogen, 34, 41, 43: serum amyloid A-I, 36: retinol binding protein, 37, 39: C4b-binding protein alpha chain, 38: gelsolin, 39: human serum albumin, 42: serum amyloid A-IV, 43: haemoglobin β-component, 45: complement C4, 54: fibrinogen alpha chain].

These two fractions were then mixed to make a single fraction of pH range 5.4 to 7.0. After protein precipitation, 2-DGE of this fraction was performed on IPG strip of pH range 4 to 7 followed by electrophoretic separation. Master gel image was created by combining the spots from both AML and healthy gels (Fig. 3a). That was used for comparison of AML gel with healthy gel image (Fig. 3a and b). The comparison between healthy and AML samples is shown in the scatter plot (Fig. S5). Out of 182 spots, 42 spots were having a 4X quantity difference, while 137 spots were with significance level ≥95%. The spots which were with 4X quantity difference and ≥95% significant both were 41. Altogether, 182 gel spots from the control and the AML samples, when analysed by MALDI-TOF MS, led to the identification of 34 distinct proteins and/or their respective isoforms and subunits (Fig. 4). The list of identified proteins with details such as theoretical and experimental pI and molecular weight, MASCOT score, sequence coverage, etc. is shown in Table 1. A sample Mascot Score Histogram of hemopexin protein is given in supplementary section (Fig. S6).

**Table 1:** List of 34 identified proteins through mass spectrometry and MASCOT database searching.

Spot ID	Accession no.	Protein name	Score	Expect value	Matched peptides no.	Sequence coverage (%)	Molecular weight (M_r_)	Isoelectric pH (pI)
17, 53	gi|224053	Alpha-2-macroglobulin	120	3.1e-07	38/146	29%	162072	5.92
10, 51, 55	CFAH_HUMAN	Complement factor H	145	6.4e-11	45/136	35%	143680	6.21
7, 8, 52	CERU_HUMAN	Ceruloplasmin	92	1.4e-05	19/107	26%	122983	5.44
25	CFAB_HUMAN	Complement factor B	98	3.3e-06	20/90	30%	86847	6.67
24	C1R_HUMAN	Complement C1r subcomponent	75	6.7e-04	16/64	21%	81606	5.82
23, 40	THRB_HUMAN	Prothrombin	115	6.4e-08	18/88	34%	71475	5.64
37, 39	C4BPA_HUMAN	C4b-binding protein alpha chain	109	2.5e-07	19/131	39%	69042	7.15
26, 45	gi|401871713	Chain C, Complement C4 In Complex with Masp-2	80	0.003	12/104	38%	33737	6.37
2	A1BG_HUMAN	Alpha-1β-glycoprotein	83	9.5e-05	18/125	35%	54790	5.56
4	VTDB_HUMAN	Vitamin D-binding protein	79	2.5e-04	15/111	41%	54526	5.40
1, 39, 46	HEMO_HUMAN	Hemopexin	130	2e-09	19/127	41%	52385	6.55
28, 29, 48	gi|223170	Fibrinogen gamma chain	76	7.5e-03	10/66	31%	46823	5.54
3, 20	gi|37748641	Kininogen 1	112	2e-06	17/115	44%	48954	6.29
16, 35, 44	HPT_HUMAN	Haptoglobin	114	8e-08	15/102	36%	45861	6.13
18	FINC_HUMAN	Fibronectin	64	0.0079	43/179	18%	266052	5.46
22	CD5L_HUMAN	CD5 antigen-like	112	1.3e-07	17/80	55%	39603	5.28
6, 9	gi|90108664	Chain A, Apolipoprotein A-I	135	9.9e-09	15/77	62%	28061	5.27
11	SAMP_HUMAN	Serum amyloid P-component	70	2.e-03	9/76	29%	25485	6.10
5	gi|14719497	Chain A, Transthyretin	78	4.8e-03	6/81	66%	12671	5.26
15	APOA4_HUMAN	Apolipoprotein A-IV	92	1.1e-05	15/72	38%	45371	5.28
12, 13, 14, 32	APOE_HUMAN	Apolipoprotein E	112	1.3e-07	16/92	50%	36246	5.65
5	TTHY_HUMAN	Transthyretin	57	0.04	4/95	48%	15991	5.52
30, 31, 47	FIBB_HUMAN	Fibrinogen beta chain	184	8e-15	28/108	60%	56577	8.54
21	CO7_HUMAN	Complement component C7	68	0.0034	17/91	23%	96550	9.06
54	gi|11761629	Fibrinogen alpha chain precursor	83	0.0016	23/147	36%	70227	8.23
43	HBB_HUMAN	Haemoglobin subunit beta	44	0.72	6/78	49%	16102	6.75
42	SAA4_HUMAN	Serum amyloid A-4	61	0.016	6/60	40%	14851	9.17
33, 49, 50	PLMN_HUMAN	Plasminogen	183	1e-14	27/112	39%	93247	7.04
38	GELS_HUMAN	Gelsolin	46	0.46	13/105	17%	86043	5.90
34, 41, 43	SAA1_HUMAN	Serum amyloid A-1	64	0.0079	5/70	46%	13581	6.28
36	RET4_HUMAN	Retinol-binding protein	53	0.11	9/63	51%	23337	5.76
24	C1S_HUMAN	Complement C1s subcomponent	48	0.29	18/113	25%	78174	4.86
27	gi|374977533	Alpha-1-microglobulin	79	0.0037	10/117	63%	22030	6.25
39	ALBU_HUMAN	Serum albumin	94	7.7e-06	21/129	39%	71317	5.92

Spot IDs are those mentioned in Fig. 4.

Using PDQuest, we further analysed the 2-DGE spots to identify most significant and consistently dysregulated 15 proteins in AML cases (showing ≥4-fold increase/decrease in spot intensity and with significance level more than 95% in *t*-test), in comparison to the control subject. The results unequivocally showed variability in the levels of the SAA1, HPT, complement factor B, CD5 antigen-like, kininogen-1, fibrinogen gamma chain, C4b-binding protein alpha chain, complement factor 7, ApoA1, ApoE, plasminogen, apolipoprotein A-IV, prothrombin, fibronectin, and gelsolin. The former 6 proteins were found to be up-regulated, while the latter 9 were down-regulated in the AML, as shown in column graphs (Fig. S7).

### Gene ontology (GO) analysis

Based on the known or postulated biological functions of the proteins as found in the GO consortium using homo sapiens taxon, the functions of the identified 34 proteins could be categorized on the basis of their functions as binding (28%), enzyme regulation activity (7%), homeostasis (7%), structural (2%), receptor mediated activity (15%), catalytic (14%), biological process regulation (25%) while for 2% proteins the functions are not yet known (Fig. S8).

### Validation through ELISA

**Figure 5:**
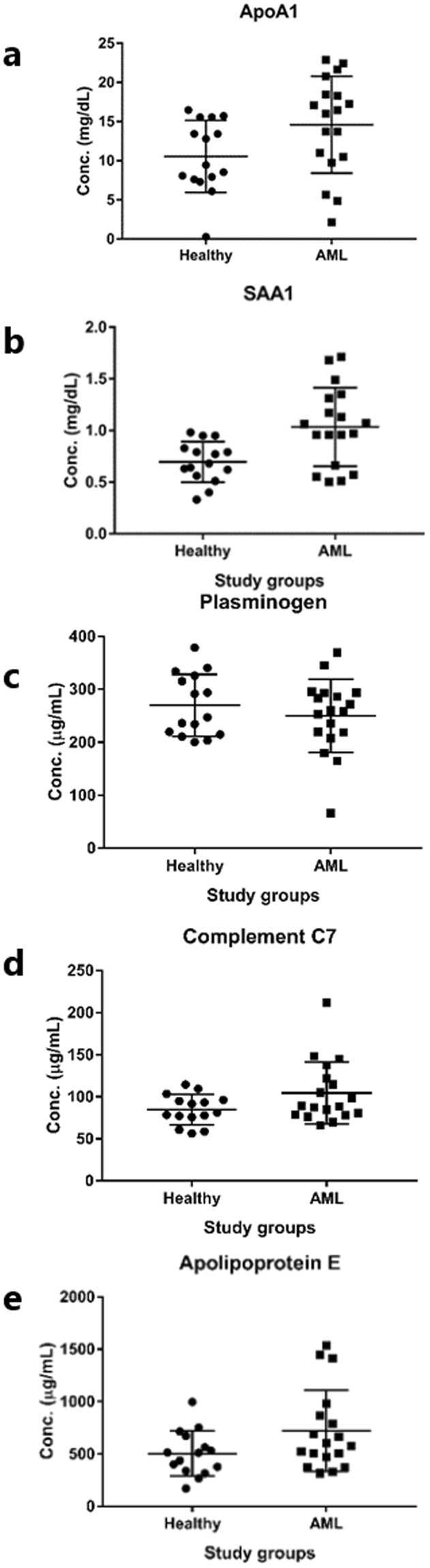
Scatter plot with standard deviation achieved through ELISA results of individual samples of healthy and AML subjects. (**a**) ApoA1 protein, (**b**) SAA1 protein, (**c**) plasminogen, (**d**) complement factor C7, (**e**) apolipoprotein E.

ApoA1 was found to be down-regulated in AML pool (10.67 mg/dL), in agreement with the spot intensity in the 2-DGE image of AML, as compared to the healthy pool (18.15 mg/dL). However, when the individual samples of AML were analysed, the mean plasma concentration of ApoA1 in the healthy group was 10.55 ± 4.6 mg/dL, whereas in the AML subjects it was found to be 14.59 ± 6.2 mg/dL *i.e*., higher, quite contrary to the expected results (Fig. 5). We applied *t*-test with Welch’s correction (One-way ANOVA) to compare the variances. The mean concentrations were found to be significantly different with *p* < 0.0396, while the variances were not significantly different in individual samples.

SAA1 was found to be up-regulated in AML pool sample (1.15 mg/dL), in comparison to the pool sample of healthy individuals (0.89 mg/dL). Validation results of individual samples also showed significant differences when validated through ELISA. The mean plasma concentration of SAA1 in the individual samples of control was 0.69 ± 0.19 mg/dL whereas it was found to be 1.04 ± 0.38 mg/dL *i.e*., high in the AML subjects (Fig. 5). Mean concentrations were found to be significantly different with *p* < 0.0029, and the variances were also significantly different in individual samples with *p* < 0.0162 by applying statistical analysis.

Plasminogen was found to be down-regulated in AML patients in comparison to the healthy individuals through ELISA validation and found to agree with 2-DGE results. When pool samples of AML and healthy individuals were compared, the value of plasminogen was found to be low in AML (216.42 µg/mL), in comparison to healthy pool (244.87 µg/mL). Similarly, the mean plasma concentration of plasminogen in healthy and AML individuals was 269.8 ± 58.74 µg/mL, and 250.1 ± 69.31 µg/mL, respectively (Fig. 5). Statistical analysis showed significant difference of mean concentrations with *p* < 0.3844, and the variances with *p* < 0.5372.

In AML complement factor 7 (C7) protein was found to be up-regulated in comparison to healthy individuals, contrary to 2-DGE results, where we found C7 was down-regulated in AML pool gel, in comparison to healthy pool gel. Although not a big difference, the complement C7 protein value in healthy pool was 94.68 µg/mL versus 98.09 µg/mL in AML pool. Plasma mean value was 84.62 ± 17.92 µg/mL in healthy, while 104.6 ± 36.96 µg/mL in AML patients (Fig. 5). *T*-test with Welch’s correction showed no significant difference between AML and healthy with *p* value 0.0537, while *F*-test to compare variances showed significant difference among study groups with *p* value 0.0090.

ApoE was found to be up-regulated in AML in contrast to 2-DGE results. ApoE value in healthy pool was 517.79 µg/mL versus AML pool was 564.82 µg/mL. Mean value in AML patients was found to be 504.4 ± 214.2 µg/mL, versus 720.2 ± 387.5 µg/mL in healthy group (Fig. 5). *T*-test with Welch’s correction gave no significant difference with *p* value 0.0532, and when variances were compared through *F*-test, a significant difference with *p* value 0.0300 was observed.

## Discussion

The combination of 2-DGE and mass spectrometry offers a powerful tool to investigate the proteomic expression profiles to identify biomarkers which might serve as indicators of the disease^27^. Recently many potential protein biomarkers have been reported in the literature for the diagnosis of AML e.g. ApoE, complement factor H, HPT, apolipoprotein A-N, SAA1, and gelsolin using proteomics techniques^28–31^. Also various MS based studies have already been done on cell lines^32–35^. In this study, we have investigated differential proteomic profile pattern among AML and healthy samples in plasma by employing multi-dimensional fractionation strategy to identify biomarker proteins.

The major bottleneck in analysing plasma proteins is to analyse low abundant proteins in the presence of high abundant proteins^36^. Therefore, to overcome this difficulty, we first depleted the samples for high abundant proteins to unmask the proteins which are present in very low amount. The depletion column with antibodies for the top 7 abundant proteins, including albumin, fibrinogen and HPT were used. While doing depletion, we have noticed that 100% depletion of plasma samples was not achieved, because in flow-through (unbound fraction), we have also identified peaks of albumin, all three chains of fibrinogen (α, β and γ), and HPT (Fig. 4). In terms of specificity of immunodepletion, contaminants in bound and eluted portion have been already reported by manufacturers and researchers^37^. Albumin may be present due to non-specific interactions with other proteins in the plasma, which is called the “sponge-effect”^37–40^. HPT and three chains of fibrinogen were visible in unbound fraction possibly due to the narrow-range techniques applied, or the fragments may have low binding efficiency to the column. The presence of target proteins (to be depleted through column) as contaminants have already been reported^22,41^.

After depletion, many low abundant proteins were visualized by IEF. In-solution IEF eases the detection of low abundance proteins and increases the detection range as large amounts of proteins of specific pH can be loaded on the gel^13^. In 2-DGE maps, most of the identified proteins were represented by multiple spots in AML and control group (Fig. 4). Slight to moderate shifts in pI or mass were between theoretical and experimentally-calculated values has already been reported in most of the cases. Both observations *i.e*., representation of single protein by multiple spots and variations in theoretical and experimentally calculated pI/MW, seem to be the result of post-translational modifications, especially glycosylation, affecting the electrophoretic mobility of the proteins, as reported earlier^42^.

**Figure 6:**
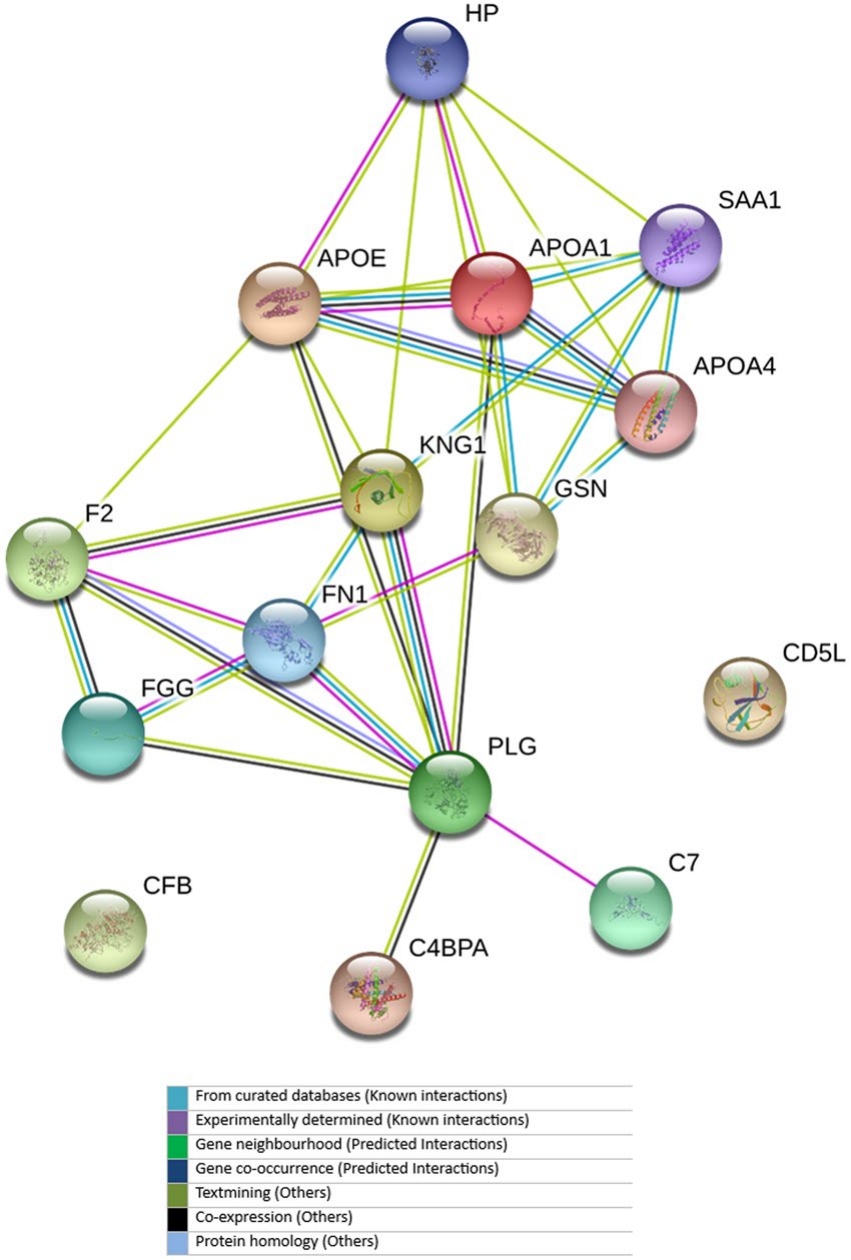
Curated pathway of fifteen differentially expressed proteins in acute myeloid leukaemia acquired from online STRING database. Balls with structures show that their 3D structures are also available in database.

To gain an insight of the biological functions and interactive links that are known to be associated with the differentially expressed proteins in the dataset, STRING software program was used. Interactive links between 15 proteins of the dataset could be traced as presented in Fig. 6. Blue lines show phylogenetic co-occurrence between proteins, whereas light blue lines represent database evidence. The line thickness is a rough indicator of the power of the association. The visualizations among protein nodes show the predicted association between the proteins detected in the samples of AML and non-leukemic healthy patients. The pathways involved by these differentially regulated proteins are presented in Table S3.

We further validated some of the deregulated proteins through ELISA, as it has the potential to accelerate validation of protein biomarkers for clinical use^43^. HPT and all three chains of fibrinogen were not included for the validation because depletion column had affinity for HPT and fibrinogen, and most of them had depleted out of plasma sample in column, so these results seen in 2-DGE was not reliable.

SAA1 is a major acute phase reactant and is also found as apolipoprotein of the HDL complex. ELISA validation results of SAA1 show similar trend as 2-DGE results, and hence SAA1 may be used as a potential diagnostic biomarker for AML (Fig. 5). Plasminogen; Plasmin dissolves the fibrin of blood clots and acts as a proteolytic factor in a variety of other processes including, embryonic development, tissue remodelling, tumour invasion, and inflammation. Plasminogen was found to be down-regulated in ELISA validation.

ApoA1; participates in the reverse transport of cholesterol from tissues to the liver for excretion by promoting cholesterol efflux from tissues and by acting as a cofactor for the lecithin cholesterol acyltransferase (LCAT), and as part of the SPAP complex activates spermatozoa motility. The results from the analysis of ApoA1 protein from individual samples were in contradiction from the 2-DGE maps (Fig. 5). Therefore, these results of ApoA1 protein require further validation on large number of individual samples.

Complement component 7; constituent of the membrane attack complex (MAC) plays a key role in the innate and adaptive immune response by forming pores in the plasma membrane of target cells. ApoE mediates the binding, internalization, and catabolism of lipoprotein particles. It serves as a ligand for the LDL (ApoB/E) receptor, and for the specific ApoE receptor (chylomicron remnant) of hepatic tissues. Results of both complement C7 and ApoE were not in agreement with 2-DGE results.

**Figure 7:**
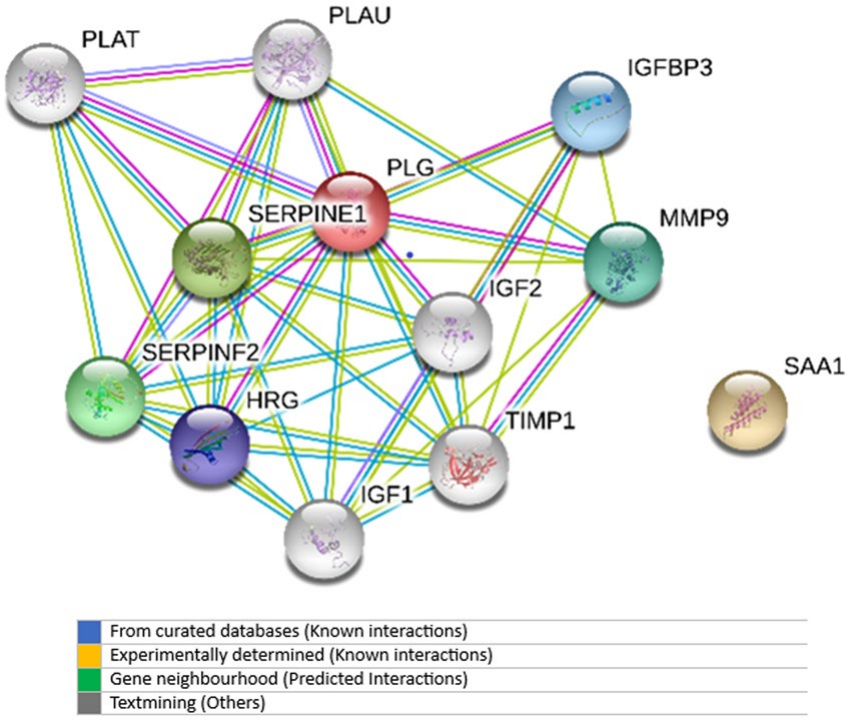
Pathways analysis of SAA1 and plasminogen.

During ELISA validation, we found that out of five differentiating proteins, only two proteins SAA1 and plasminogen showed potential of differentiation of AML from healthy group, during ELISA validation. These two proteins showed links to 4 Kyoto Encyclopedia of Genes and Genomes (KEGG) pathways including transcriptional misregulation in cancers, p53 signalling pathway, proteoglycans in cancers, and hypoxia-inducible factor 1 (HIF-1) signalling pathway (Fig. 7). These pathways regulate cell proliferation and apoptosis, inflammation, metabolism, angiogenesis, cell growth and migration, cell migration and metastasis, cell cycle arrest, cellular senescence, and DNA repair, which are properties of cancerous cell. Therefore, the relevant up-regulation of SAA1 and down-regulation of plasminogen may be due to the direct effect of these disturbed signalling pathways, and can be used for the diagnosis of acute myeloid leukaemia in future.

## Conclusion

In conclusion, 5D proteomic strategy using immunodepletion, 2-DGE, ZOOM-IEF and MALDI-MS, and ELISA analysis has shown a promising approach for the detection of differentiated proteins in AML in comparison with the control. Fifteen proteins were found to be deregulated in comparison to healthy control. Some of these deregulated proteins were further validated through ELISA technique and the results suggest that SAA1 and plasminogen can be used as biomarkers for the diagnosis of AML patients. However, as the validation was performed on a small number of proteins, therefore validation of these deregulated proteins on a larger number of individual subjects is needed.

## Electronic supplementary material

Supplementary information
