# Supplementary material for: 5D proteomic approach for the biomarker search in plasma: Acute myeloid leukaemia as a case study
Source: Sci Rep. 2017 Nov 27;7:16440. doi: 10.1038/s41598-017-16699-2 (PMC5703949; doi:10.1038/s41598-017-16699-2)
Supplement: Supplementary file 1 — Supplementary information [file 41598_2017_16699_MOESM1_ESM.docx]

5D proteomic approach for the biomarker search in plasma: Acute myeloid leukaemia as a case study.

Syed Kashif Raza^1^, Mahwish Saleem^1^, Tahir Shamsi ^4^, M. Iqbal Choudhary^1, 2, 3^ and Atta-ur-Rahman^1, 2^,Syed Ghulam Musharraf* ^1, 2^,

^1^Dr. Panjwani Center for Molecular Medicine and Drug Research, International Center for Chemical and Biological Science, University of Karachi, Karachi – 75270, Pakistan.

^2^H.E.J. Research Institute of Chemistry, International Center for Chemical and Biological Science, University of Karachi, Karachi – 75270, Pakistan.

^3^Department of Biochemistry, Faculty of Sciences, King Abdulaziz University, Jeddah 21412, Saudi Arabia.

^4^National institute of Blood Diseases, Karachi, Pakistan.

**Corresponding Authors**

***** Dr. Panjwani Center for Molecular Medicine and Drug Research, International Center for Chemical and Biological Science, University of Karachi, Karachi – 75270, Pakistan

Tel.: 00-92-21-99261701-2

00-92-21-111222292 (ext. 134)

Fax: 00-92-213-34819018

00-92-99261713-4

E. mail: [musharraf1977@yahoo.com](mailto:musharraf1977@yahoo.com)

[musharraf@iccs.edu](mailto:musharraf@iccs.edu)

**Keywords**

Acute myeloid leukaemia, MALDI-TOF mass spectrometry, ZOOM-IEF, plasminogen, serum amyloid A-1, 5D proteomic

**Table S1:** Number of acute myeloid leukaemia samples and their clinical classification according to the French-American-British (FAB) at the time of sampling.

|  | **Subtypes** | **Number of patients** |
| --- | --- | --- |
| **AML** | M1 without maturation | 13 |
|  | M2 with granulocytic maturation | 18 |
|  | M3 acute promyelocytic | 7 |
|  | M4 granulocytic and monocytic maturation | 3 |
|  | M5a monoblastic | 1 |
|  | M5b monocytic | 2 |
|  | not classified at the time of sampling | 6 |

**Table S 2:** Statistics and strategy of collecting healthy plasma samples.

|  | **Healthy** | |
| --- | --- | --- |
| **Age group** | 20-65 years |  |
|  | 20-30 yrs | Group I |
|  | 30-40 yrs | Group II |
|  | 40-50 yrs | Group III |
|  | 50-65 yrs | Group IV |
| **Number of samples** | 100 | |
| **Gender** | Male (50) | |
|  | Female (50) | |

**Table S3:** Biological pathways involved by differentially regulated proteins in acute myeloid leukaemia.

| **Pathway** | **Proteins** |
| --- | --- |
| Annotated pathway (KEGG)  Name: Complement and coagulation cascades | KNG1 F2 PLG C7 FGG C4BPA and 58 other proteins |
| Curated pathway  Biochemical Reaction: Amyloid precursor proteins form ordered fibrils | APOA1 SAA1 APOA4 GSN and 17 other proteins |
| Curated pathway  Pathway: Amyloids | APOA1 SAA1 APOA4 GSN and 19 other proteins |
| Curated pathway  Pathway: Chylomicron-mediated lipid transport | APOA1 APOE APOA4 and 13 other proteins |
| Curated pathway  Biochemical Reaction: NREH hydrolyses atREs (HSPG:apoE) to atROL and FAs | APOA1 APOE APOA4 and 15 other proteins |
| Curated pathway  Biochemical Reaction: LRPs transport extracellular CR:atREs:HSPG:apoE to cytosol | APOA1 APOE APOA4 and 15 other proteins |
| Curated pathway  Complex: CR:atREs:HSPG:apoE | APOA1 APOE APOA4 and 15 other proteins |
| Annotated pathway (KEGG)  Name: Regulation of actin cytoskeleton | F2  FN1  GSN  and 198 other proteins |
| Curated pathway  Catalysis: LRPs transport extracellular CR:atREs:HSPG:apoE to cytosol | APOA1 APOE APOA4 and 18 other proteins |

**Figure S 1:** Chromatogram of fast protein liquid chromatography (FPLC) during depletion of top seven abundant proteins using MARS hu-7 column. Red lines and number show the fractions; fraction 1 is before the unbound fractions, fraction 2 is unbound portion (AML and healthy pool), and fraction 3 is bound fraction (AML and healthy pool).


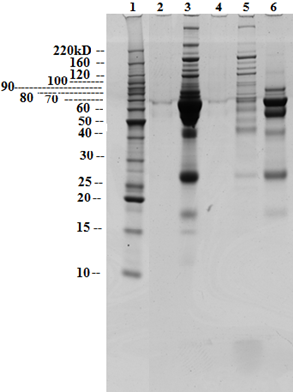


**Figure S 2:** Checking depletion efficiency of MARS column. Fractions loaded were equivalent to 0.1 µL original crude human plasma on 12 % SDS-PAGE.1: Protein ladder 2: Blank 3: Crude plasma (AML pool) 4: Fraction-1, i.e. before elution of unbound portion (AML pool) 5: Fraction-2, unbound portion (AML pool) 6: Fraction-3, bound portion (AML pool).


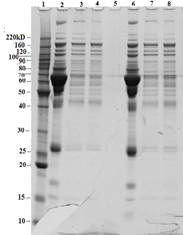


**Figure S 3**: Checking enrichment efficiency of Fraction-2 unbound fraction by 5Kda MWCO tubes. Fractions loaded were equivalent to 0.1 µL original crude human plasma on 12 % SDS-PAGE 1: Protein ladder, 2: Crude plasma (Healthy pool) 3: Fraction-2, unbound portion (Healthy pool) before enrichment 4: Fraction-2, unbound portion (Healthy pool) afterenrichmente 5: Blank 6: Crude plasma (AML pool) 7: Fraction-2, unbound portion (AML pool) before enrichment 8: Fraction-2, unbound portion (AML pool) after enrichment.


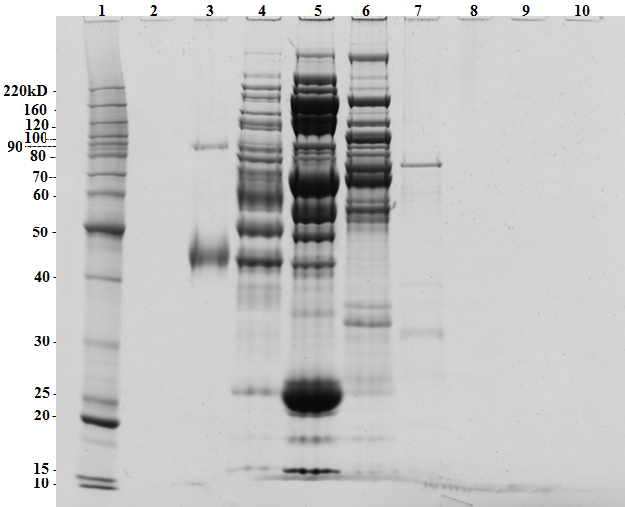


**Figure S 4:** IEF fractions after ZOOM_IEF of healthy pool. 15 µL (Max load) of each fraction was loaded onto 12%, 10 well’s gel. 1: Protein ladder 2: Blank 3: Fraction-1 healthy pool (pH 3.0-4.6) 4: Fraction-2 healthy pool (pH 4.6-5.4) 5: Fraction-3 healthy pool (pH 5.4-6.2) 6: Fraction-4 healthy pool (pH 6.2-7.0) 7: Fraction-5 healthy pool (pH 7.0-10.0.


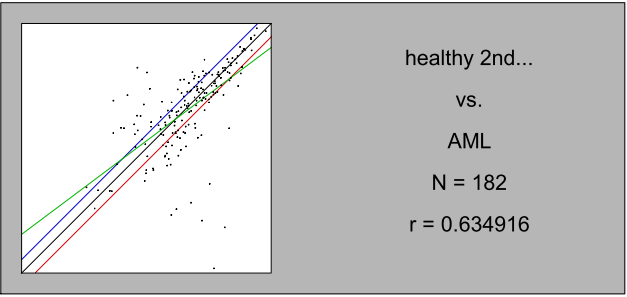


**Figure S 5:** Scatter plot of spots between healthy and AML pool gels.


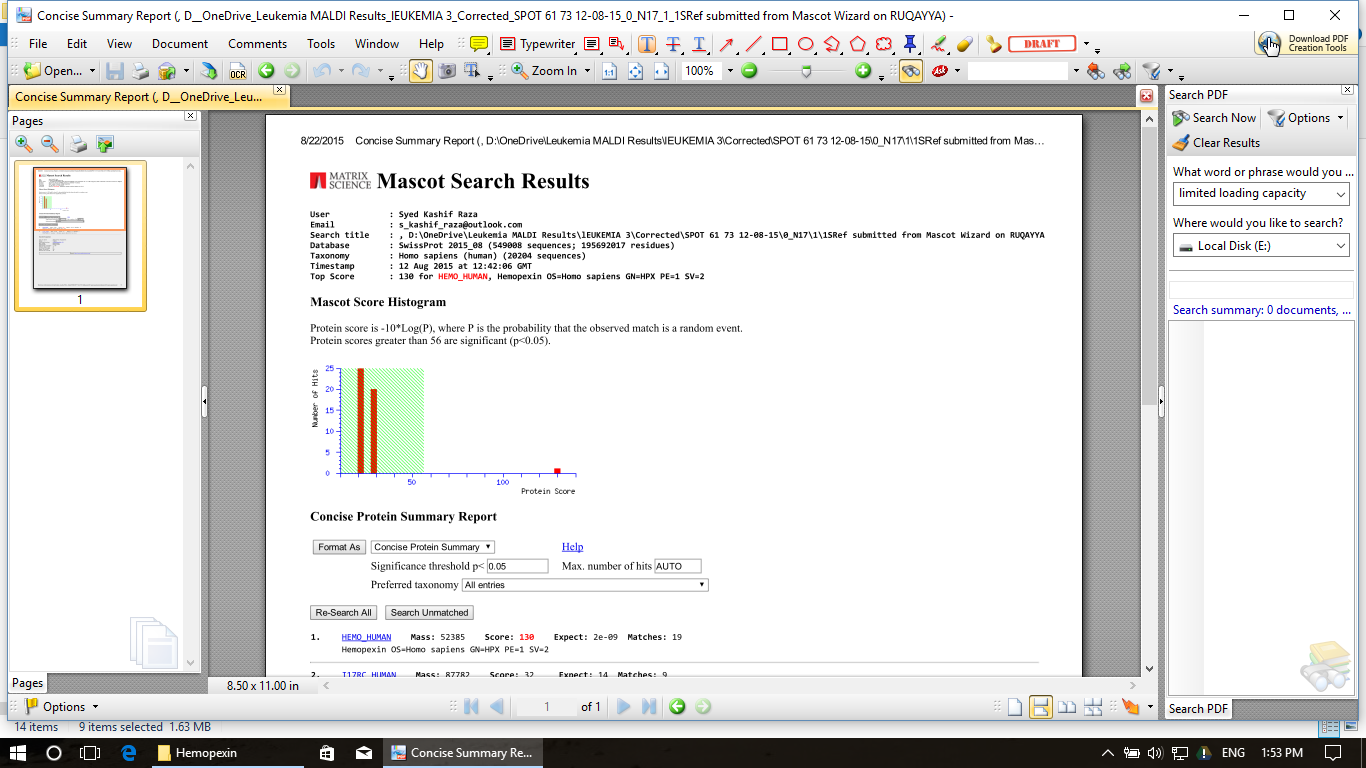


**Figure S 6:**Mascot Score Histogram of hemopexin. Individual ions scores >56 indicate identity or extensive homology (p < 0.05). Protein scores are derived from ions scores as a non-probabilistic basis for ranking protein hits.


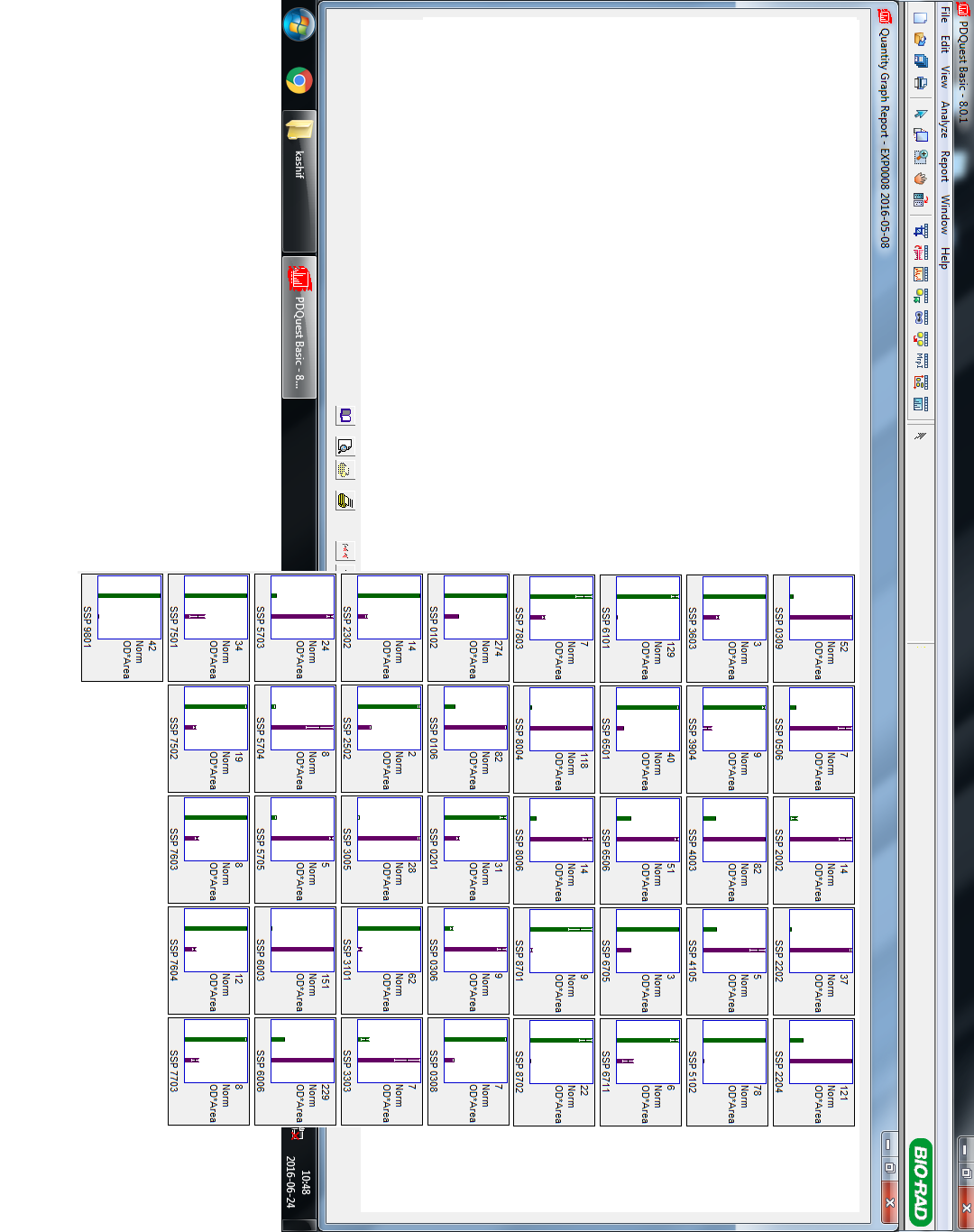


**Figure S 7:**Quantity graph report of differentially expressed proteins in the unbound portion of AML and healthy pool. Green colour is for healthy and purple colour is for AML. Spots number are those, which are highlighted in Figure 3 with green crosses assigned by PDQuest software.

**Figure S 8:** Protein functional categories of identified 34 proteins based on gene ontology consortium using homo-sapiens taxon.

**Unprocessed Figures**

| **a** | **b** |
| --- | --- |
| 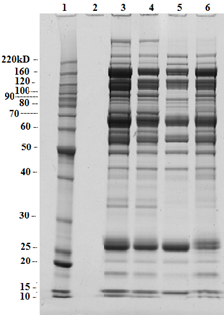 | 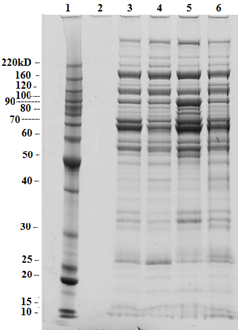 |

**Figure 2:a:**Comparison of fraction-3 (pH: 5.4-6.2) after ZOOM-IEF. 1: Protein ladder 2: Blank 3: APA 4: MDS 5: Healthy 6: AML,**b:** Comparison of fraction-4 (pH: 6.2-7.0) after ZOOM-IEF. 1: Protein ladder 2: Blank 3: APA 4: MDS 5: Healthy 6: AML
